# Synthesizing Molecular Insights to Redefine the Battle Against Metastatic Cancer

**DOI:** 10.3390/cancers18020199

**Published:** 2026-01-08

**Authors:** Babak Behnam

**Affiliations:** 1 Biology Department, College of Art and Sciences, American University, Washington, DC 20016, USA; bbehnam@american.edu or babak.behnam@gmail.com; 2 Floret Advanced Genomics & Bioinformatics Research Center, Lagos 100271, Nigeria

## 1. Introduction: The Frontier of Metastatic Biology

Metastasis represents the most formidable challenge in clinical oncology, accounting for over 90% of cancer-related deaths. This grim statistic underscores a persistent therapeutic gap: while we have made substantial progress in managing primary tumors, our ability to prevent or cure disseminated disease remains profoundly limited. This failure stems not from a lack of effort but from the extraordinary biological complexity of the metastatic cascade, a multi-step process involving dynamic cellular plasticity, adaptive survival mechanisms, and sophisticated hijacking of host systems. This Special Issue of *Cancers* directly addresses this complexity, assembling a series of investigations that dissect metastasis from the level of single molecules to systemic pathophysiology. The studies presented extend beyond the classical linear model of progression and toward an integrated understanding of metastasis as a systemic, evolutionarily adaptive disease. This Editorial synthesizes these insights, arguing that the path to transformative clinical breakthroughs lies in connecting these diverse molecular discoveries to create a new paradigm for interception and combination therapy.

This Special Issue transforms a segmented view of metastasis into an integrated model. The story is no longer solely about a cancer cell activating a single pathway to migrate. Instead, it is about a cell that dynamically rewires its signaling (AKT2, TCF12) (Contribution 4), remodels its own architecture and waste systems (vesicular trafficking) (Contribution 7), manipulates its energy sources and surroundings (TSGA10, FMD) (Contributions 6), and masterfully deceives the immune system (MACC1, T-cell duality) (Contribution 10) to complete its lethal journey.

The most promising future lies at the intersection of these layers. Could targeting endosomal trafficking impair the secretion of immune-suppressive signals like PD-L1? Might metabolic therapies that enhance TSGA10 function simultaneously starve a tumor and expose it to immune attack (Contribution 9)? Emerging paradigms, such as ghost mitochondria, serve as a crucial reminder that metastasis may be fueled by biological processes that we are only just beginning to describe.

## 2. An Integrated Overview: From Cellular Hijacking to Systemic Dysregulation

The contributions collectively reveal metastasis not as a single pathway but as a constellation of interconnected biological capabilities, each presenting unique vulnerabilities. The emerging portrait is of a cell that is metabolically agile, immunologically elusive, and capable of extraordinary phenotypic adaptation.

### 2.1. Mastery of Cellular Plasticity and Signaling Networks

At its core, metastasis is an exercise in cellular reprogramming. The epithelial-to-mesenchymal transition (EMT) remains a cornerstone of this plasticity, conferring invasive properties and therapy resistance. However, current research reveals EMT as a dynamic spectrum rather than a binary switch, regulated by intricate post-transcriptional and post-translational networks. The control of key EMT transcription factors like ZEB1 by ubiquitin ligases exemplifies how protein stability mechanisms are recruited to drive phenotypic fluidity (Contribution 2) [1]. Beyond EMT, new dimensions of plasticity have emerged, including the formation of polyploid giant cancer cells (PGCCs) (Contribution 8), stress-resistant entities that can spawn aggressive, genetically variant progeny through a primitive, depolyploidization cycle, contributing to tumor heterogeneity and relapse [2].

This plasticity is fueled by the precise rewiring of canonical signaling pathways. The PI3K-AKT-mTOR axis, a central regulator of growth and survival, demonstrates isoform-specific functionality in metastasis. For instance, AKT2, but not AKT1, has been shown to be specifically required for the metastatic dissemination of BRAF-mutant melanoma, where it regulates a distinct gene signature linked to glycolysis and invasion (Contributions 4 & 5) [3]. This underscores a critical theme: effective therapeutic targeting may require moving beyond pan-pathway inhibition toward disrupting specific molecular subroutines commandeered for dissemination. Furthermore, metastasis involves the creation of self-sustaining signaling loops. For example, the transcription factor TCF12 can directly activate the expression of TGF-β2, creating an autocrine/paracrine circuit that sustains both EMT and the immunosuppressive tumor microenvironment, thereby linking developmental pathways directly to metastatic progression (Contribution 5) [4].

### 2.2. Metabolic Reprogramming and Ecosystem Engineering

To survive the metabolically challenging journey from the primary site to a distant organ, disseminating tumor cells (DTCs) undergo profound metabolic reprogramming. This is not a passive response but an active, instructional program that enables every step of the metastatic cascade. A key orchestrator is the hypoxia-inducible factor (HIF) family, which drives a shift toward glycolysis (the Warburg effect) even in the presence of oxygen, while also promoting angiogenesis and invasion [5]. This metabolic shift generates biosynthetic precursors and manages oxidative stress, allowing cells to thrive in adverse conditions.

However, this metabolic addiction also presents a profound therapeutic vulnerability. Strategies like caloric restriction mimetics and fasting-mimicking diets (FMDs) exploit this by inducing a severe, selective metabolic stress on cancer cells while protecting normal tissues (Contribution 3). Crucially, as highlighted in this Special Issue, such interventions can synergize powerfully with chemotherapy by inhibiting pro-survival autophagy and, more importantly, by potentiating anti-tumor immunity through mechanisms involving T-cell-dependent clearance [6]. This demonstrates a fundamental principle: the intrinsic metabolism of the tumor cell, systemic host metabolism, and the immune response are inextricably linked nodes in the metastatic network.

A particularly fascinating frontier is the role of intercellular metabolic hijacking. Emerging evidence points to the direct transfer of organelles, most notably mitochondria, between cells in the tumor microenvironment. Cancer cells can use tunneling nanotubes (TNTs) to “steal” functional mitochondria from neighboring stromal cells, thereby replenishing their bioenergetic capacity and enhancing their metastatic fitness [7]. Conversely, they may donate dysfunctional mitochondria to immune cells, such as cytotoxic T lymphocytes, to impair their anti-tumor function (Contribution 11), a remarkable example of cellular piracy that blurs the line between metabolic and immune evasion strategies [8].

This strategic co-option of host resources underscores a broader repertoire of emergent, non-genetic paradigms that enable metastasis. As summarized in Table 1, these advanced adaptations; ranging from organelle hijacking and the formation of polyploid giant cancer cells to the sculpting of alternative vascular networks (Contribution 8), represent fundamental shifts in our understanding of tumor biology. Critically, each paradigm reveals a distinct therapeutic vulnerability. Moving from mechanistic insight to clinical translation requires a focused effort to develop agents that can disrupt these specific processes, such as inhibiting tunneling nanotube formation to block mitochondrial trafficking or targeting the unique metabolic dependencies of polyploid cells. Thus, the table not only catalogs these emerging frontiers but also maps them directly to potential intervention strategies, highlighting a roadmap for the next generation of anti-metastatic therapeutics.

### 2.3. Subversion of the Immune System and Systemic Niches

Metastasis is unequivocally a systemic disease. The primary tumor does not merely shed passive cells into circulation; it actively manipulates the entire host to create a receptive environment for its progeny. A cornerstone of this manipulation is the formation of the pre-metastatic niche (PMN). Through secreted factors (like VEGF, TGF-β, and PTHrP) and extracellular vesicles (exosomes), the primary tumor “educates” distant organs, such as the lungs, liver, or bone marrow [9]. This education involves recruiting bone marrow-derived cells (e.g., myeloid-derived suppressor cells or MDSCs), remodeling the extracellular matrix, and increasing vascular permeability, all to create a supportive landing pad for circulating tumor cells.

Central to both PMN formation and the survival of established metastases is immunosuppression. Metastatic cells evade immune destruction through a multi-layered strategy: upregulating immune checkpoint ligands (e.g., PD-L1), secreting factors that recruit regulatory T cells (Tregs) and MDSCs, and expressing non-classical MHC molecules. Molecules like MACC1 have been identified as central hubs in this process, driving invasive growth while simultaneously upregulating PD-L1 and other immunosuppressive factors, thereby directly linking the mechanisms of motility and immune escape (Contribution 10) [10]. The immune landscape itself exhibits a critical duality, as seen in the context of breast cancer, where T lymphocytes can be polarized toward pro-tumorigenic (Th2, Treg) or anti-tumorigenic (Th1, cytotoxic CD8+) phenotypes, with the balance dramatically influencing metastatic outcomes (Contribution 11) [11].

### 2.4. The Logistical Machinery of Spread

Beneath these high-order programs lies the critical, often overlooked, logistics of dissemination. The endosomal–lysosomal vesicular trafficking system emerges as a master regulator of the metastatic cascade (Contribution 7). Motor proteins like dynein and kinesins do not merely transport cargo; they direct the polarized delivery of integrins, matrix metalloproteinases (MMPs), and growth factor receptors to the leading edge of invading cells [12]. Furthermore, this machinery is essential for the secretion of exosomes, which act as long-range messengers to prepare pre-metastatic niches. By delivering oncogenic proteins, lipids, and nucleic acids (like miRNAs and circRNAs), tumor-derived exosomes can reprogram recipient cells in distant organs to support future colonization [13]. Targeting this logistical hub, for instance via inhibiting specific motor proteins or disrupting exosome biogenesis, represents a promising strategy to cripple both local invasion and systemic communication.

## 3. Conclusions: Charting a Path Toward Interceptive and Integrative Therapeutics

The collective narrative emerging from this Special Issue is one of convergence. Metastasis is no longer seen through the narrow lens of a single “driver” mutation or pathway but is understood as the integrated output of appointed plasticity, metabolism, and immune evasion programs, all orchestrated by a cell with remarkable adaptive intelligence. This refined understanding dismantles the old paradigm of treating metastasis as a late-stage complication and replaces it with an urgent imperative for early interception and systemic disruption.

The future of anti-metastatic therapy therefore lies in rational, mechanism-based combinations that attack the process on multiple fronts simultaneously (Contribution 1). The insights herein suggest several strategic pillars for this new therapeutic architecture:

(A) Therapies Targeting Adaptive States—Combating plasticity requires agents that can lock cells into a more vulnerable, differentiated state or eliminate plastic subpopulations (e.g., PGCCs, EMT hybrid cells). This may involve targeting epigenetic readers/writers, key transcription factor complexes, or stress-survival pathways like autophagy that permit transitions between states.
(B) Metabolic Warfare—Exploiting the metabolic dependencies of metastatic cells, especially during the vulnerable phases of dissemination and colonization, involves combining dietary interventions (e.g., FMD) with drugs that target oxidative phosphorylation, glutamine metabolism, or lipid synthesis. A key frontier disrupts the metabolic crosstalk between tumor cells and stromal cells in the niche.
(C) Disruption of Systemic Communication—Neutralizing the tools tumors use to engineer their own spread is a viable prophylactic strategy. This could involve inhibitors of exosome secretion, neutralization of key niche-educating factors (e.g., LOXL2, MFGE8), or blockers of tunneling nanotube formation to prevent mitochondrial hijacking.
(D) Immunotherapy 2.0 [14] via Re-educating the Ecosystem—Emphasizes combining modalities actively reprogram the metastatic microenvironment rather than relying on checkpoint blockade alone. This includes agents that deplete or reprogram MDSCs and TAMs, vaccines targeting metastasis-associated antigens, and adoptive cell therapies engineered to overcome metabolic suppression in the niche.
(E) Advanced Biomarkers for Interception—To deploy these strategies effectively, we must detect the systemic process of metastasis earlier. The future lies in “liquid biopsy” 2.0 [15], extending beyond simple mutation detection in ctDNA to analyzing the phenotype of circulating tumor cells (CTCs), the cargo of exosomes, and immune cell profiles to assess the real-time activity of the metastatic cascade and the state of the pre-metastatic niche.

In conclusion, this Special Issue provides essential molecular and conceptual information for assembling a new, more effective war against metastatic cancer. By integrating the principles of cellular piracy, metabolic symbiosis, and ecological manipulation, we are building a unified theory of metastasis that is both more complex and more target-rich than ever before. The challenge is no longer purely biological; it is an engineering challenge of designing intelligent, timed, and layered therapeutic combinations. By embracing this integrative and interceptive paradigm, we can transform the clinical trajectory of metastatic disease from one of inevitable progression to one of durable control and prevention. This is the decisive frontier in oncology, and this Special Issue’s research paves the way forward.

To translate this knowledge into clinical progress, the field must embrace combinatorial strategies. The future of anti-metastatic therapy likely rests on rational drug combinations that concurrently target a cancer cell’s intrinsic drive to disseminate and its extrinsic ability to hide and thrive. The articles in this Special Issue provide a robust scientific foundation for building these strategies. By continuing to dissect the molecular mechanisms with the depth and breadth showcased here, we move closer to the ultimate goal: transforming metastasis from a terminal event into a controllable process.

## Figures and Tables

**Table 1:** Emerging metastatic paradigms and their therapeutic implications.

Paradigm	Core Mechanism	Potential Therapeutic Strategy
Mitochondrial Hijacking	Transfer of functional mitochondria to cancer cells via tunneling nanotubes (TNTs) to boost fitness.	Inhibiting TNT formation (e.g., via M-Sec inhibition); blocking mitochondrial uptake.
Immune Cell Sabotage	Donation of dysfunctional mitochondria from cancer cells to T cells to impair effector function.	Enhancing mitochondrial quality control in T cells; metabolic support for immunotherapy.
Polyploid Giant Cancer Cells (PGCCs)	Stress-induced polyploidization leading to depolyploidization and generation of aggressive, resistant progeny.	Targeting the depolyploidization process (e.g., Aurora kinases); exploiting vulnerabilities of PGCCs (e.g., autophagy dependence).
Vasculogenic Mimicry	Formation of perfusable, matrix-rich channels by tumor cells, independent of endothelial cells, to support perfusion.	Disrupting tumor cell plasticity programs or targeting key matrix modifiers (e.g., LOXL2).
Metabolic-Epigenetic Coupling	Oncometabolites (e.g., 2-HG, fumarate) directly inhibiting or activating epigenetic enzymes to lock in pro-metastatic gene expression.	Targeting mutant metabolic enzymes (IDH1/2 inhibitors); using epigenetic drugs to reverse dysregulated gene programs.

## References

[1] Zhang P., Wei Y., Wang L., Debeb B.G., Yuan Y., Zhang J., Yuan J., Wang M., Chen D., Sun Y. (2014). ATM-mediated stabilization of ZEB1 promotes DNA damage response and radioresistance through CHK1. Nat. Cell Biol..

[2] White-Gilbertson S., Voelkel-Johnson C. (2020). Giants and Monsters: Unexpected Characters in the Story of Cancer Recurrence. Adv Cancer Res..

[3] Chin Y.R., Yoshida T., Marusyk A., Beck A.H., Polyak K., Toker A. (2014). Targeting Akt3 signaling in triple-negative breast cancer. Cancer Res..

[4] Sánchez-Tilló E., Liu Y., de Barrios O., Siles L., Fanlo L., Cuatrecasas M., Darling D.S., Dean D.C., Castells A., Postigo A. (2012). EMT-activating transcription factors in cancer: Beyond EMT and tumor invasiveness. Cell. Mol. Life Sci..

[5] Semenza G.L. (2010). HIF-1: Upstream and downstream of cancer metabolism. Curr. Opin. Genet. Dev..

[6] Di Biase S., Lee C., Brandhorst S., Manes B., Buono R., Cheng C.W., Cacciottolo M., Martin-Montalvo A., de Cabo R., Wei M. (2016). Fasting-Mimicking Diet Reduces HO-1 to Promote T Cell-Mediated Tumor Cytotoxicity. Cancer Cell.

[7] Dong L.F., Kovarova J., Bajzikova M., Bezawork-Geleta A., Svec D., Endaya B., Sachaphibulkij K., Coelho A.R., Sebkova N., Ruzickova A. (2017). Horizontal transfer of whole mitochondria restores tumorigenic potential in mitochondrial DNA-deficient cancer cells. eLife.

[8] Saha T., Dash C., Jayabalan R., Khiste S., Kulkarni A., Kurmi K., Mondal J., Majumder P.K., Bardia A., Jang H.L. (2022). Intercellular nanotubes mediate mitochondrial trafficking between cancer and immune cells. Nat. Nanotechnol..

[9] Peinado H., Zhang H., Matei I.R., Costa-Silva B., Hoshino A., Rodrigues G., Psaila B., Kaplan R.N., Bromberg J.F., Kang Y. (2017). Pre-metastatic niches: Organ-specific homes for metastases. Nat. Rev. Cancer.

[10] Korshunov A., Sahm F., Zheludkova O., Golanov A., Stichel D., Schrimpf D., Ryzhova M., Potapov A., Habel A., Meyer J. (2019). DNA methylation profiling is a method of choice for molecular verification of pediatric WNT-activated medulloblastomas. Neuro Oncol..

[11] DeNardo D.G., Brennan D.J., Rexhepaj E., Ruffell B., Shiao S.L., Madden S.F., Gallagher W.M., Wadhwani N., Keil S.D., Junaid S.A. (2011). Leukocyte complexity predicts breast cancer survival and functionally regulates response to chemotherapy. Cancer Discov..

[12] Steffan J.J., Dykes S.S., Coleman D.T., Adams L.K., Rogers D., Carroll J.L., Williams B.J., Cardelli J.A. (2014). Supporting a role for the GTPase Rab7 in prostate cancer progression. PLoS ONE.

[13] Hoshino A., Costa-Silva B., Shen T.L., Rodrigues G., Hashimoto A., Tesic Mark M., Molina H., Kohsaka S., Di Giannatale A., Ceder S. (2015). Tumour exosome integrins determine organotropic metastasis. Nature.

[14] Morad G., Helmink B.A., Sharma P., Wargo J.A. (2021). Hallmarks of response, resistance, and toxicity to immune checkpoint blockade. Cell.

[15] Heitzer E., Haque I.S., Roberts C.E.S., Speicher M.R. (2019). Current and future perspectives of liquid biopsies in genomics-driven oncology. Nat. Rev. Genet..

